# Boston Letter

**Published:** 1882-02

**Authors:** 


### Domestic Correspondence.

### ARTICLE XII.

### BOSTON LETTER.

MESSRS. EDITORS:—It is somewhat doubtful whether the new building for the Harvard Medical School will be ready for occupancy before next winter. Three stories have already been erected. The location is excellent. The present school building stands in a squalid portion of the town which must have surprised many a visitor. The new structure is located in the midst of the best quarter of the city, having fine churches, public buildings and private dwellings of the better sort in its immediate neighborhood. It is midway between the Massachusetts General and City Hospitals, to both of which students resort for clinical instruction. The new school building, therefore, will in many ways, aside from modern improvements, offer increased conveniences to teachers and students. Surgical operations will be done, and clinical surgery and clinical medicine will, as now, be taught in the two hospitals. Practical pathology will likewise be taught in a building specially constructed for the purpose in the grounds of the Massachusetts General Hospital. Dissection, however, will be carried on in the new building. Three hundred thousand dollars have already been raised to defray the expenses of construction.

The pathological building to which I have referred is probably unique. Its peculiarity consists in the details of the apartment in which the post mortems are made. It need not be said that the one difficulty in this feature of medical instruction is created by the formerly unavoidable odor from the cadaver when under the knife. This objection applies principally to first year men.

In order to vanquish this objection, Dr. Henry J. Bigelow brought his singularly apt ingenuity to bear, and his experiments resulted in perfect success.

The principal object being the disposal of foul gases, Dr. Bigelow determined to set in action, in some way, a series of draughts; and knowing that when a cadaver is opened the disagreeable odors arise chiefly from the trunk, he saw that the draught must act not only upon this section of the body, but must act *downward*. His conception resulted in a table of iron gradually depressed toward the center. It rests upon a hopper-shaped box, also of iron, supported in its turn by a cylinder which telescopes into the head of a larger cylinder, in which, near the floor, it revolves by means of a shoulder. Table and hopper are one. The former is accurately covered by a movable apron of zinc or oxydized copper, having in its center an opening measuring 20 by 28 inches, and corresponding to the position occupied by the trunk of a subject.

Into this opening fits a concave grating of polished steel. The hopper and the double cylinder,—the latter extending into the cellar below,—have a porcelain lining. In the basement, immediately under the center of the table, is the trapped opening of a large drain, which receives all fluids and such small solid matters as can pass through the meshes of the grating, the hopper and cylinder being much larger than the grating in the table. But it is the gases and odors for which this arrangement was conceived. Their escape requires a description of the most ingenious feature of the whole. Some feet down the cylinder, and nearly half way to its lower end, is a large aperture mouthing into a lateral shaft placed just below the floor of the post-mortem room. This shaft runs horizontally, and terminates fifteen feet away in a special chimney which passes upward through a small room adjoining the autopsy department. In this ante-room is a window let into the chimney. Opening the window, you find in the chimney a series of ten gas jets. Lighting these, and waiting a few moments for the warming of the air in the chimney, you hold a bit of burning paper over the opening in the table, and are surprised by the force with which the flame is drawn *down* the hopper. And this is the manner in which, during the examination of a body, gases and odors leave the room, its air remaining pure. A similar arrangement, on a smaller scale, is applied in an upright glass case with sliding sash, which encloses the sink, and is always kept shut. Herein, too, are gas-burners and a connection with another flue. During the examination, cleansing, or temporary disposal of an organ, this sink is used, and all odors are thus carried away. Hot and cold water serve the convenience of the examiner.

The autopsy table is supplied with every convenience to secure cleanliness, a small hose being the chief. The floor of the room is of asphalt, the washboards are glass, so that the whole place is frequently deluged with water, which runs off by means of scuppers and gratings which communicate with the main drain. Near the table is a glass case furnished with instruments of every sort; there is a large ice chest for the preservation of specimens; also a convenient room devoted to the preparation of specimens and microscopic work, and many other conveniences which render the place as nearly perfect as one can well imagine. The building is a neat structure of brick. The amphitheater is of moderate size, finished in chestnut, and abundantly supplied with light and warmth. Can you do better than to imitate this room in Chicago?

In the same district, and not far from the new building of the Harvard Medical School, a hospital for children is also in process of construction. The corner stone was laid June 13, 1881. The land embraces 31,000 feet. The building will cost $100,000, and will have every modern improvement. The hospital heretofore has occupied a large dwelling-house, but has outgrown its quarters. Thus far 1,590 patients have been treated, the institution being thirteen years old. The medical and surgical staffs are composed of some of our best men, and the interest of the laity in this hospital is warm and constant. The hospital has a very efficient auxiliary in the Ladies' Aid Association. They not only take upon themselves the entire care and support of the Convalescent Home of the hospital, but contribute largely to the latter in services, money and effects. When these buildings are finished you shall have a description of them.

I am happy to announce the publication of a new book by a

Boston physician. This is not so common an occurrence with us as to be unworthy of mention. Indeed, a volume of five hundred pages from the pen of one of our physicians but rarely appears in Boston. Our men are workers, hard workers in fact, but their pens are less prolific than those of physicians in other cities; why, it would be difficult to say. It is a Boston peculiarity. The author of the book to which I refer is Dr. Henry W. Williams; its title "Diseases of the Eye." It is charmingly written, is free from many of those technicalities which render works on the eye such dull reading to the general practitioner, and its subjects are presented with such simplicity of style and clearness of diction, that it will become a necessity even to those who do not make the eye a specialty. In short, to make use of that threadbare, hackneyed, but convenient phrase, the book "supplies a want long felt." Dr. Williams is professor of ophthalmology in the Harvard Medical School, and president of the Massachusetts Medical Society. He is well known as a distinguished oculist, and no physician of our city has warmer friends than he.

Another event which the members of our profession naturally regard with a feeling of pride, is the election of Dr. Samuel A. Green to the office of mayor. A fair indication of his character and of the esteem in which he is held, is the fact that Dr. Green is not a party, but a *citizen's* candidate. He entered upon his duties unfettered by promises to men or party, and his acts during the first fortnight of his administration evince an independence which, in these degenerate days of party influence, is truly refreshing. For years Dr. Green has been city physician, and his deeds of unselfish kindness and faithful performance of duty will not soon be forgotten. He has been one of the overseers of the poor, whilom chairman (without salary) of the Public Library, and in other and various ways he has shown a devotion to the interests of the city which makes his record almost exceptional.

A sad experience coming upon us within a few days has been the loss by death of Drs. Thomas B. Curtis, John Bacon and Edward Reynolds. Dr. Curtis was president of the Boston Society for Medical Improvement. His death was sudden and unexpected. Although but thirty-nine years of age, Dr. Curtis was considered one of the most brilliant and learned of the physicians of Boston. Among the men of his years, he had no peer in his scholarly knowledge of medicine. His loss is irreparable. The society over which he presided have decided upon a portrait in oil, to be hung in the society hall as a fitting and deserved memorial of their lamented president.

Dr. John Bacon was less active in the medical world. Some years ago he retired from the chair of chemistry in the Harvard Medical School. Since that time he has lived a life of such seclusion as to have become almost forgotten in the onward rush of the lives of busier men. He died at the age of seventy years.

Dr. Edward Reynolds was a genial and most lovable old gentleman. Formerly very active, a pupil of the great French and English teachers of sixty years ago (Astley Cooper, William Lawrence, Dupuytren and Bichat), he was one of the first men who ever lectured on surgery in the East. In conjunction with Jacob Bigelow, D. H. Storer and O. W. Holmes, he originated and for many years taught surgery in the Tremont Medical School. He established the Massachusetts Eye and Ear Infirmary in 1815, and its success was due almost entirely to him alone. As a *raconteur* he possessed most enviable qualities. Two years ago, being then eighty-six years of age, Dr. Reynolds one evening entertained one of our medical societies with reminiscences of deceased colleagues and former friends. The easy flow of his language, the polish of his style, his ready and fertile memory, his apt and elegant choice of words, his eloquence and wit, were as surprising as they were fascinating. Those who listened to him on this occasion will not soon lose the memory of the charm of his diction and delivery. Until the end his mind was clear, and he died the quiet, beautiful death of a beloved old man, of a physician who lived a long, earnest, fruitful, faithful life. His son, Dr. John P. Reynolds, is professor of obstetrics in the medical department of Harvard University.

BOSTON, Jan. 12, 1882. * *

